# Vaccination Strategies: Mixing Paths Versus Matching Tracks

**DOI:** 10.3390/vaccines13030308

**Published:** 2025-03-13

**Authors:** Achilleas Livieratos, Charalambos Gogos, Iason Thomas, Karolina Akinosoglou

**Affiliations:** 1 Independent Researcher, 15238 Athens, Greece; 2 Department of Medicine, University of Patras, 26504 Rio, Greece; cgogos@med.upatras.gr (C.G.); akin@upatras.gr (K.A.); 3 Allergy Centre, Wythenshawe Hospital, Manchester University Hospitals NHS Foundation Trust, Manchester M23 9LT, UK; iason.thomas@manchester.ac.uk; 4 Faculty of Biology, Medicine and Health, University of Manchester, Manchester M1 7HR, UK; 5 Department of Internal Medicine and Infectious Diseases, University General Hospital of Patras, 26504 Rio, Greece

**Keywords:** heterologous vaccination, homologous vaccination, SARS-CoV-2, adaptive immunity

## Abstract

Vaccination strategies play a pivotal role in achieving broad and robust immune protection. With the advent of new technologies and challenges posed by emerging infectious diseases such as SARS-CoV-2, evaluating the efficacy of homologous (matching tracks) and heterologous (mixing paths) vaccination regimens is critical. This article explores mechanistic insights and empirical evidence on the benefits and limitations of these approaches.

## 1. Introduction

Vaccination remains a cornerstone of public health due to its unparalleled ability to stimulate protective immune responses and prevent the spread of infectious diseases. The immune system is commonly activated through a prime-boost strategy where a primary dose prepares the immune cells, and a booster enhances and extends the immune response [1,2]. Homologous vaccination regimens, which use the same vaccine type for both doses, have been the standard [1,2]. For instance, the mRNA immunization against SARS-CoV-2 has demonstrated sustained efficacy through homologous boosting by maintaining high antibody levels and consistent immune activation [1,2]. Homologous regimens simplify logistics and have been extensively tested for safety and effectiveness, providing a stable foundation for immunization campaigns [1,2]. However, this approach faces limitations, including reduced efficacy against emerging variants and potential waning immunity over time. These limitations are particularly visible during pandemics, like SARS-CoV-2, whereby healthcare professionals and regulators must respond swiftly and effectively.

Heterologous regimens involve combining different vaccine platforms to enhance immune responses [1,2]. Specifically, this refers to the administration of the same vaccine for the first two doses, followed by a different vaccine platform for the booster dose [1,2]. For example, using an adenovirus vaccine platform as a primer, which is then immunized afterwards by a booster like Pfizer-BioNTech’s BNT162b2, has demonstrated significantly greater immunogenicity compared to homologous adenovirus schedules [1,2]. Studies reveal that heterologous combinations elicit up to 20–60-fold higher neutralizing antibody titers (nAb) and stronger T-cell responses [1,2]. By combining the distinct mechanisms of action from different platforms, such as the robust antibody production of mRNA vaccines and the strong cellular responses induced by viral vectors, heterologous regimens represent a promising evolution in vaccine strategy. Vulnerable populations stand to benefit the most from evolving vaccination strategies, particularly in the face of emerging pandemics.

## 2. Unmet Needs and Emerging Challenges: Patients with Dysregulated Immune Responses

Modern vaccination strategies currently face the critical challenge of addressing a growing subset of the population with dysregulated immune systems. This group includes the elderly, immunocompromised individuals, and those with chronic diseases, all of whom often exhibit reduced vaccine efficacy [3]. For example, influenza vaccines have demonstrated highly variable efficacy over the past few decades, ranging from 10% to 60%, primarily due to pre-existing immunity and antigenic drift [3].

A study of 377 solid-organ-transplant recipients found that homologous regimens with Pfizer-BioNTech (BNT162b2) or Moderna (mRNA-1273) led to very low seroconversion rates [4,5]. About 52% achieved antibody production after the second dose, compared to higher responses (63%) with heterologous regimens involving mRNA and viral vector vaccines [4,5]. Another study, however, showed that ChAdOx1 (AstraZeneca) regimens failed to elicit sufficient antibody titers in transplant patients, with only 55% achieving detectable antibody responses even after a third dose [5,6]. Furthermore, homologous vaccination with viral vector vaccines like Sputnik V or AstraZeneca in autoimmune patients resulted in a strong immune response, with 95% of patients achieving adequate neutralizing antibody levels versus all patients via heterologous immunization [4,5]. Finally, homologous mRNA vaccine regimens resulted in a neutralizing antibody response rate of just 19% in a cohort of 97 solid-organ-transplant patients [4,6]. Comparatively, heterologous regimens yielded higher rates (50%) [4,6].

These challenges have called for innovative approaches, including heterologous vaccination regimens—combining platforms such as mRNA and viral vectors—that have shown great promise in enhancing immune responses in these vulnerable populations [5,6].

## 3. Mechanistic Insights into Immunization Strategies

### 3.1. Homologous Prime-Boost Mechanisms

Homologous prime-boost vaccination relies on repeated exposure to the same vaccine antigen to reinforce and extend immune memory [7,8]. For example, mRNA immunization stimulates the adaptive immune response by encoding the spike peptides, inducing a strong neutralizing humoral response after a second dose [7,8]. This repeated exposure enhances the activation of B cells, leading to the formation of memory cellular immunity in the lymph nodes [7,8]. The second dose also promotes the expansion of CD4+ T helper cells, particularly those secreting interferon-γ (Th1-skewed cellular population) and tumour necrosis factor, which supports robust antibody production. CD8+ cytotoxic T cells are moderately activated, targeting infected cells for destruction [7,8].

However, homologous regimens face challenges such as reduced efficacy against new viral variants [7,8]. For instance, homologous ChAdOx1 (AstraZeneca) regimens (though not generalizable to mRNA homologous vaccines) generate IgG and nAbs, but the quality and durability of these responses diminish over time, particularly against variants like Omicron [7,8]. Furthermore, repeated administration of the same viral vector vaccine may induce anti-vector immunity, reducing the efficacy of subsequent doses [7,8].

### 3.2. Heterologous Prime-Boost Mechanisms

Heterologous regimens combine different vaccine platforms, such as a viral vector prime followed by an mRNA booster, to optimize the immune response [7,8]. These regimens exploit the unique strengths of each vaccine type [7,8]. For instance, viral vectors like ChAdOx1 induce strong T-cell responses, especially CD8+ cytotoxic T cells, which are crucial for intracellular pathogen clearance [7,8]. When boosted with an mRNA vaccine, the response is amplified through robust antibody production, driven by activated memory B cells and Th1-helper cells [7,8]. This combination elicits complementary and durable immunity, involving both cellular (T cells) and humoral (antibody-mediated) defences [7,8].

Mechanistically, heterologous regimens may prevent immune exhaustion observed in homologous schedules [7,8]. DNA vaccines, for example, serve as excellent primers by presenting antigens directly to antigen-presenting cells (APCs) like dendritic cells, effectively priming naïve T cells [7,8]. Boosting with a protein or mRNA vaccine then reinforces this response, significantly enhancing antibody titers and memory T-cell populations [7,8]. Studies have shown that DNA-prime and protein-boost approaches for diseases like tuberculosis result in significantly higher IgG avidity and expanded T follicular helper (Tfh) cell responses compared to homologous protein–protein regimens [7,8].

**Figure 1:**
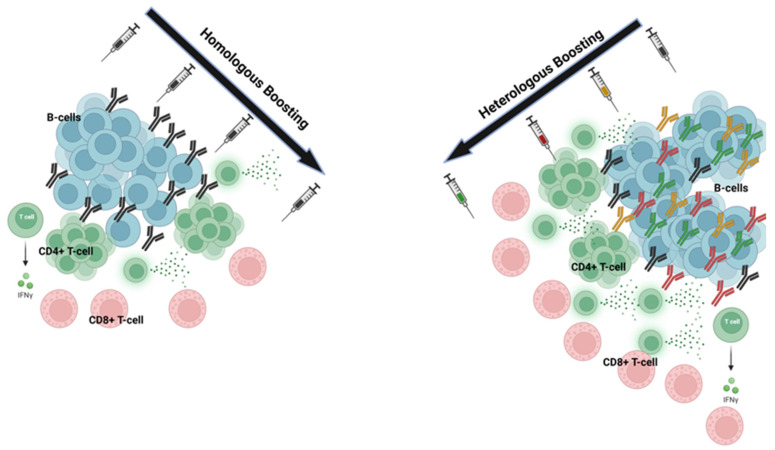
Immune profile after homologous and heterologous immunization.

These mechanisms highlight the immunological advantages of heterologous prime-boost strategies, such as waning immunity and reduced efficacy against variants [9,10,11,12,13,14]. The interplay between cellular and humoral immunity in these approaches underscores their potential to offer broad and durable protection against pathogens (Figure 1).

Figure 1: Expanded cellular and humoral immunity across different viral variants during heterologous boosting. In homologous boosting, repeated doses of the same vaccine primarily activate B-cells with a moderate CD4+ T-cell response and CD8+ T-cell activation, resulting in a relatively uniform antibody profile. Heterologous boosting combines different vaccine platforms, leading to greater diversity in B-cell responses, strong activation of CD4+ and CD8+ T-cells, and a strong production of interferon-gamma (IFN-γ).

### 3.3. Heterologous Immunization Outcomes

Heterologous vaccination regimens utilize different vaccine platforms, such as viral vectors, mRNA, and protein subunits, to achieve stronger and broader immune responses [15]. As sequential exposure to antigens delivered via distinct platforms enhance the activation of both T and B cells, it has been demonstrated that combining viral vector and mRNA vaccines resulted in SARS-CoV-2 incidence rates, at 25.7%, compared to homologous regimens (28.7%) [15]. As alternating vaccine platforms helps mitigate anti-vector immunity, individuals who received the Janssen vaccine followed by an mRNA booster reported stronger humoral immunity compared to those on homologous mRNA regimens [16]. A study in the UK, for example, highlighted that adolescents receiving heterologous regimens combining BNT162b2 (Pfizer) with NVX-CoV2373 (a protein-subunit vaccine) experienced enhanced immunogenicity compared to those on homologous mRNA schedules [16]. Studies have shown that combining ChAdOx1 (adenoviral vector) with mRNA vaccines results in a 33% increase in protection against symptomatic infections compared to homologous regimens [17]. This increase is largely attributed to a stronger activation of CD8+ T cells, which are critical for long-term immunity and viral clearance [17]. Supporting these findings, a Bangladeshi study reported elevated anti-spike IgG levels (13.4, antibody levels) in individuals receiving heterologous boosters, significantly surpassing (7.92 antibody levels) homologous regimens [5]. Finally, the global withdrawal of ChAdOx1 in 2024 has since reshaped heterologous immunization strategies, shifting reliance toward mRNA, the protein subunit, and inactivated vaccines [8,13].

## 4. Role of Amino-Acid-Sequence Differences in Vaccines

Differences in the amino-acid sequences among vaccines significantly influence how the immune system recognizes and responds to the virus [7,8]. As viruses continuously evolve, variants like Delta and Omicron have developed mutations in the spike protein, particularly in the receptor-binding domain, which can reduce the effectiveness of neutralizing antibodies [7,8]. To counteract these changes, strategies such as using full-length spike sequences enhance immunogenicity [7,8]. The method of delivery also plays a role, as mRNA vaccines encode a stabilized spike protein that closely mimics the virus’s natural form, while viral vector vaccines use DNA to instruct cells to produce the antigen [7,8]. These differences impact how the immune system recognizes the spike protein, which directly affects vaccine efficacy against emerging viral strains [7,8].

The variation in spike-protein sequences also affects immune evasion and the immune response generated by different vaccines [7,8]. Changes in the sequence can modify antigen presentation by determining which peptide fragments are displayed, thereby impacting T-cell activation [7,8]. As a result, the strength and duration of memory B-cell and antibody responses are also impacted [7,8]. Incorporating updated spike sequences or using heterologous vaccination can enhance immunity by exposing the body to a broader array of targets [7,8]. This strategy has proven beneficial against viral variants, where mixed-vaccine regimens trigger stronger T-cell responses, counteracting immune escape observed in homologous vaccination [7,8].

## 5. Pre-COVID-19 Evidence and Insights Generated

Before the COVID-19 pandemic, heterologous vaccination strategies were extensively explored across various infectious diseases, demonstrating their potential to enhance immunogenicity and address limitations associated with homologous regimens.

In the case of Ebola vaccines, a prime-boost strategy utilizing adenovirus vectors followed by modified vaccinia Ankara (MVA) platforms achieved remarkable success. Clinical trials reported 90% immunogenicity, indicating that this heterologous approach was highly effective in eliciting robust and sustained immune responses [18]. This demonstrated the advantages of combining different vaccine platforms to enhance protection against severe viral infections.

For HIV, heterologous vaccination strategies also showed promise. Sequential regimens involving DNA-based priming followed by vector-based boosting were found to significantly enhance both B- and T-cell responses. Such responses are critical for combating HIV, a pathogen characterized by its rapid mutation and immune evasion strategies [19]. These findings highlighted the ability of heterologous regimens to induce broad and durable immunity, which is crucial for addressing the challenges posed by variable viruses.

Heterologous regimens were similarly effective in improving immune responses to influenza vaccines. Studies on H3N2 influenza revealed that heterologous approaches elicited stronger mucosal and systemic immune responses compared to homologous vaccination [3]. This is particularly important for respiratory pathogens, as mucosal immunity plays a key role in preventing infection at the site of entry while systemic immunity provides broader protection [3].

The evidence from these pre-COVID-19 applications underscores the versatility and efficacy of heterologous vaccination strategies. They have been especially valuable for individuals with reduced immune responsiveness, such as the elderly or immunocompromised patients, as well as for combating pathogens that require multifaceted immune responses. The lessons learned from these studies laid a strong foundation for the adoption of heterologous immunization strategies during the COVID-19 pandemic, enabling enhanced vaccine efficacy and broader population-level protection.

## 6. The COVID-19 Case Study

The European Medicines Agency (EMA) has assessed homologous vs. heterologous immunization strategies, to evaluate their immunological effectiveness and safety [20]. The EMA found that heterologous vaccination regimens, which involve using different vaccine platforms for the initial and booster doses, can enhance immune responses compared to homologous vaccination (Table 1) [20]. This approach was particularly useful for increasing flexibility in vaccine deployment and addressing concerns related to adverse events associated with certain vaccines [20]. Heterologous combinations, such as viral vector vaccines followed by mRNA vaccines, led to stronger antibody and T-cell responses, including increased neutralizing activity and broader immune coverage against variants [20]. While the primary focus of EMA’s review was COVID-19 vaccination, heterologous vaccination strategies have historically been applied to other infectious diseases, suggesting broader potential utility.

### 6.1. Comparative Long-Term Efficacy of Heterologous Versus Homologous Regimens

During the COVID-19 pandemic, heterologous vaccination regimens emerged as an effective strategy to address vaccine shortages, while enhancing immunogenicity. These regimens demonstrated significant immunological and clinical advantages compared to homologous schedules, across diverse populations and vaccine combinations.

Heterologous boosters using mRNA vaccines provided significantly higher antibody titers compared to homologous regimens based on inactivated or vector-based vaccines [15]. Notably, patients receiving the BNT162b2 vaccine as a heterologous booster achieved the highest antibody levels post-third dose, outperforming combinations involving ChAdOx1 (AstraZeneca) or CoronaVac [5].

A study conducted across 10 sites in the United States reinforced the immunological benefits of heterologous regimens. Participants receiving heterologous boosters exhibited neutralizing antibody titer increases ranging from 6-fold to 73-fold, compared to 4-fold to 20-fold for homologous regimens [17]. Importantly, individuals primed with adenovirus vector vaccines and boosted with mRNA vaccines experienced a 55-fold increase in binding antibody titers, highlighting the synergistic effects of mixed-vaccine platforms [17].

Real-world evidence from the Vision Network further supported the clinical effectiveness of heterologous regimens. The study reported a 79% vaccine efficacy (VE) against emergency care visits for heterologous regimens, compared to 54% VE for homologous adenoviral schedules [23]. Hospitalization rates were also significantly reduced, with 90% VE for three mRNA doses and 78% VE for regimens combining adenoviral and mRNA vaccines [23]. These findings underscore the practical benefits of mixed schedules in reducing severe COVID-19 outcomes.

Recent research highlighted the durability of immune responses with heterologous regimens, particularly against Omicron variants [15]. Participants boosted with Ad26.COV2.S demonstrated more gradual antibody declines over 16 weeks, with a 2.1-fold reduction compared to 6.9-fold for homologous schedules [24]. This durability underscores the role of heterologous regimens in sustaining immunity amid the ongoing evolution of SARS-CoV-2 variants.

Data from Vietnam revealed that heterologous regimens were associated with reduced adverse events compared to homologous Pfizer vaccinations [21]. The study reported a two-fold increase for adverse events with homologous Pfizer regimens, indicating the improved tolerability of mixed schedules [21]. These findings align with broader evidence supporting the safety and adaptability of heterologous vaccination approaches. Heterologous regimens showed also more durable immune responses, with other studies reporting slower antibody decline rates in mixed schedules (2.1-fold reduction over 16 weeks) compared to homologous regimens (6.9-fold reduction) [25].

Among immunocompromised individuals, heterologous strategies have demonstrated up to a 20% higher antibody positivity rate compared to homologous regimens [4]. Supporting this, recent data revealed that heterologous combinations, such as ChAdOx1 followed by mRNA vaccines like mRNA-1273 or BNT162b2, induced significantly higher levels of both humoral and cellular immunity [3].

### 6.2. Protection Against Emerging Variants

The adaptability of heterologous vaccination regimens has been crucial in addressing the challenges posed by emerging SARS-CoV-2 variants [26]. These regimens have demonstrated significant advantages in terms of immunogenicity, protection, and logistical flexibility compared to homologous regimens (same vaccine for all doses).

During surges of the Omicron variant, studies conducted in Latin America highlighted the superior efficacy of heterologous vaccination regimens. Specifically, these schedules provided up to 33% higher protection against symptomatic infection compared to homologous regimens, which underscores their potential in improving population-level immunity during variant outbreaks [6].

In Bangladesh, participants who received heterologous booster doses during post-delta variants exhibited a prolonged maintenance of antibody levels [5], The rate of antibody decline nearly halved compared to individuals on homologous regimens using inactivated vaccines across variants [5]. This highlights the long-term benefits of heterologous regimens, particularly in regions reliant on inactivated vaccines.

Among adolescents, a UK trial revealed that heterologous regimens incorporating protein-subunit vaccines, such as NVX-CoV2373, elicited stronger neutralizing antibody responses against Omicron variants compared to homologous mRNA regimens [27]. These findings are relevant for optimizing vaccination strategies in younger populations, who may benefit from heterologous immunization tailored to emerging variants.

A comprehensive network meta-analysis provided robust evidence of the effectiveness of heterologous regimens in reducing hospitalizations during the Delta and Omicron waves. Three-dose heterologous regimens achieved 93% efficacy in preventing hospitalizations, significantly outperforming homologous regimens, which achieved 92% efficacy [22].

A combination of adenovirus vector immunization, boosted by a mRNA dose achieved 94% efficacy against non-Delta and non-Omicron infections [22]. This was slightly higher than the 93% efficacy observed with three doses of mRNA vaccines [22]. These results highlight the importance of heterologous immunization strategies, particularly in addressing global challenges such as vaccine supply-chain constraints and inequitable distribution.

Heterologous vaccination regimens also played a pivotal role in countries facing vaccine supply constraints, such as Canada. A national survey reported that 16.4% of Canadians received heterologous doses, with the majority of participants perceiving improved immunity as a result [28]. This flexibility allowed for the efficient utilization of available vaccine stocks while maintaining high levels of protection, which is particularly important during periods when viral variants exhibit high mutation rates.

Studies demonstrated that mixing AstraZeneca (viral vector) with Moderna or Pfizer (mRNA) vaccines resulted in markedly higher neutralizing antibody titers and T-cell activation [29]. In one Spanish trial, antibody responses were 150 times higher after a second heterologous dose compared to baseline [29]. These regimens have shown robust cross-reactive immunity against variants such as Delta and Omicron, where higher binding and neutralizing antibodies were reported with Moderna as a second dose following AstraZeneca [30].

## 7. Future Directions

Studies have demonstrated that heterologous immunization strategies can enhance immunogenicity, with some combinations inducing higher antibody titers and stronger cellular responses than homologous regimens (Table 2) [31,32,33,34,35,36,37,38,39,40].

Specifically, mRNA-based boosters (e.g., Pfizer + Moderna, MOD-MOD-BNT) showed the highest antibody titers, while adenovirus vector vaccines followed by mRNA boosters (e.g., AstraZeneca + Moderna, ChAdOx1 + BNT162b2) generated stronger T-cell responses [31,32,33,34,35,36,37,38,39,40]. Some regimens, such as ChAdOx1 + mRNA-1273, induced the most robust immune response but also had the highest reactogenicity [31,32,33,34,35,36,37,38,39,40]. Studies demonstrated that heterologous regimens effectively reduced infections, with NVX-CoV2373 as a booster reducing breakthrough infections by 89%, and heterologous Ad26.COV2.S + mRNA-1273 increasing hospitalization protection to 78% compared to 67% for homologous regimens [31,32,33,34,35,36,37,38,39,40]. Safety data showed that while all combinations were generally safe, some, particularly adenoviral vector-based regimens, exhibited higher short-term side effects [31,32,33,34,35,36,37,38,39,40]. The adaptability of heterologous regimens has also been demonstrated in settings with limited vaccine availability. A study of 719 participants in Hanoi revealed that 45.76% experienced mild adverse effects, such as localized pain, with no significant differences between homologous and heterologous regimens [22]. These results underscore the safety and flexibility of mixing vaccines, particularly during mass immunization campaigns that must balance efficacy with logistical challenges.

Despite these advantages, heterologous vaccination regimens face several unresolved challenges. One major issue is the lack of standardized protocols regarding the optimal combinations, dosing intervals, and target populations [1,2]. The variability in immune responses across different vaccine platforms complicates efforts to develop universally applicable guidelines. Furthermore, while some combinations have demonstrated strong immunogenicity, others may lead to increased reactogenicity, causing concerns about safety and tolerability [1,2]. This is particularly relevant given reports of higher systemic adverse reactions in some heterologous regimens compared to homologous counterparts [1,2]. Additionally, the precise mechanisms underlying the superior immunogenicity of heterologous vaccination—particularly regarding memory B-cell and T-cell activation—remain poorly understood. This knowledge gap limits the ability to design vaccines that fully capitalize on these benefits and optimize their long-term protective effects.

Another critical challenge is the limited data on the long-term efficacy and safety of heterologous regimens, especially in special populations such as children, pregnant individuals, and the elderly. Moreover, comparative studies across different vaccine platforms remain sparse, particularly in diverse populations. Given the variability in vaccine accessibility across different regions, it is essential to ensure that heterologous strategies are adaptable to varying supply-chain dynamics.

To address these challenges, regulatory agencies must provide updated guidelines to facilitate the widespread adoption of heterologous immunization strategies across infectious diseases. Expanding research on variant-specific boosters in heterologous sequences could further enhance defenses against highly mutable pathogens like SARS-CoV-2 [1,2]. The emergence of next-generation vaccine platforms offers promising opportunities. Self-amplifying RNA (saRNA) vaccines, for instance, are being developed to generate prolonged antigen expression with lower doses, potentially enhancing immune responses while minimizing reactogenicity [42]. Furthermore, future multivalent, adjuvanted protein-subunit vaccines further complement other vaccine platforms and expand more broadly, in heterologous regimens, on the protection offered against different viral variants [2,8]. Personalized vaccine regimens represent another frontier in immunization strategies, leveraging advances in immune profiling to tailor vaccination schedules based on an individual’s immune response, age, and health status [42].

Integrating cellular immune-response assessments into vaccination strategies has significantly advanced long-term vaccine effectiveness, particularly in the context of personalized immunization approaches [43,44,45,46,47,48,49]. Although antibody levels tend to diminish over time, T-cell-mediated immunity exhibits greater durability and plays a critical role in protecting against severe illness [43,44,45,46,47,48,49]. This finding has profound implications for vaccine policy and the development of tailored vaccination protocols that account for individual variations in immune responses [43,44,45,46,47,48,49]. By applying immune monitoring, such as measuring T-cell activation markers, personalized vaccination schedules may be formulated that maximize immune protection while minimizing unnecessary booster doses [43,44,45,46,47,48,49]. This strategy is critical for vulnerable populations, including the elderly, individuals with chronic diseases, and those at higher risk of breakthrough infections due to weaker immune responses [43,44,45,46,47,48,49]. As research in this field progresses, heterologous and personalized vaccination strategies could revolutionize global immunization efforts. By addressing the current knowledge gaps, optimizing vaccine combinations, and integrating emerging technologies, the future of vaccination will likely move toward a more flexible and tailored approach, ensuring maximum protection against infectious diseases.

## 8. Conclusions

Heterologous immunization represents an innovative strategy for achieving robust, long-lasting immune protection, addressing critical public health challenges. By leveraging different vaccine platforms, this approach enhances both humoral and cellular immune responses, improving overall efficacy. It offers a promising solution to issues such as limited vaccine supply, the emergence of novel viral variants, and population-specific differences in immune responses. In particular, heterologous vaccination plays a crucial role in sustaining immunity against rapidly evolving pathogens like SARS-CoV-2, where traditional homologous regimens may fall short.

Despite these compelling advantages, several challenges must be addressed to fully harness the potential of heterologous vaccination. A deeper understanding of the immunological mechanisms driving its superior efficacy is necessary. Optimizing dosing intervals and vaccine pairings remains a priority to maximize immune responses while minimizing adverse effects. Furthermore, while preliminary data indicate that heterologous regimens are both safe and effective, long-term studies are essential to assess their durability across different age groups, health conditions, and immunocompromised populations.

To fully realize the benefits of heterologous immunization, public health agencies must adopt dynamic and flexible immunization policies. A rigid, one-size-fits-all approach may be inadequate in rapidly evolving pandemic scenarios, as demonstrated during the COVID-19 crisis. Developing adaptable guidelines that integrate heterologous regimens will enhance vaccine effectiveness and provide superior protection against emerging variants. This necessitates continuous surveillance, large-scale clinical trials, and expert-driven recommendations to ensure that the most effective combinations are deployed.

Additionally, a globally standardized digital immunization system is paramount for tracking vaccine combinations, monitoring efficacy, and informing future policy decisions. A universally recognized digital record would empower healthcare professionals to make data-driven choices regarding booster doses, ensuring seamless vaccine administration worldwide. By embedding these strategies within national and global health frameworks, heterologous vaccination can emerge as a cornerstone in pandemic preparedness and epidemic response, fortifying global resilience against infectious diseases.

## Figures and Tables

**Table 1:** Comparison of vaccine platforms.

Platform	Advantages	Disadvantages	Examples in Homologous and Heterologous Strategies	References
mRNA (e.g., Pfizer, Moderna)	High efficacy, strong immune response, rapid adaptability to variants	Cold storage requirements, potential for myocarditis in young males	Homologous: Pfizer–Pfizer, Moderna–Moderna; Heterologous: Pfizer–AstraZeneca	Pardo et al., 2024 [4]; Adnan et al., 2024 [5]
Viral Vector (e.g., AstraZeneca, J&J)	Long-lasting immunity, no need for ultra-cold storage	Rare risk of blood clots, lower efficacy against some variants	Homologous: AstraZeneca–AstraZeneca; Heterologous: AstraZeneca–Pfizer, AstraZeneca–Moderna	Hung et al., 2023; [21] Garza-Silva et al., 2024 [6]
Protein Subunit (e.g., Novavax)	Established platform, fewer side effects	Requires adjuvant, slower production	Homologous: Novavax–Novavax; Heterologous: Novavax–mRNA	Kelly et al., 2023 [16]
Inactivated Virus (e.g., Sinopharm, Sinovac)	Well-studied platform, good safety profile	Lower efficacy, may require additional boosters	Homologous: Sinopharm–Sinopharm; Heterologous: Sinopharm–Pfizer, Sinovac–Moderna	Au et al., 2022 [22]

**Table 2:** Outcomes of heterologous vs. homologous SARS-CoV-2 immunization.

Vaccine Combination	Study (First Author, Year)	Population (n)	Immunogenicity	Efficacy	Safety	Improved over Homologous?
Pfizer + Moderna	Adnan et al., 2024 [5]	Bangladeshi university cohort (606)	Higher antibody levels with heterologous boosting	mRNA vaccines showed the highest immunogenicity	Safe, but waning immunity noted	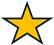
AstraZeneca + Moderna	Hung et al., 2023 [21]	Hanoi, Vietnam (719)	Comparable immunogenicity to homologous AstraZeneca	Safe, with mild adverse events in 45.8% of participants	No major safety concerns	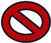
Gam-COVID-Vac + Pfizer	Garza-Silva et al., 2024 [6]	Mexico and Argentina (491)	High antibody titers maintained after six months	Effective, with protection comparable to homologous schemes	Safe, though moderate adverse events increased after booster	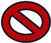
BNT162b2 + NVX-CoV2373	Kelly et al., 2023 [16]	UK adolescents (148)	NVX induced stronger T-cell response and comparable antibody levels	NVX reduced breakthrough infection risk by 89% compared to BNT-30	Safe, with lower reactogenicity in BNT-10 recipients	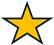
MOD-MOD-BNT	Baglivo et al., 2023 [15]	Southern Italy (469,069)	Heterologous boosting provided highest protection	Most effective in reducing Omicron infection	Safe, with lower infection risk than homologous	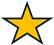
mRNA-1273 + Ad26.COV2.S	Atmar et al., 2022 [17]	US adults (458)	Heterologous boosting increased neutralizing antibody titers 6-73x	Higher T-cell responses compared to homologous boosting	Safe, though reactogenicity similar to primary series	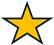
Adenovirus Vector + mRNA	Au et al., 2022 [22]	Global meta-analysis (193,955,736)	94% effectiveness against non-Delta/Omicron infections	Effective against hospitalization with OR 0.06 (95% CI: 0.02–0.21)	Safe, with effectiveness comparable to homologous three-dose regimens	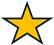
BNT162b2 + Ad26.COV2.S	Tan et al., 2022 [24]	US cohort study (68)	Heterologous Ad26 boosting provided more durable antibody and T-cell responses	Effective against Omicron with sustained immunity over 16 weeks	Safe, with similar reactogenicity to homologous boosting	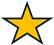
ChAdOx1 + BNT162b2	Klemis et al., 2022 [25]	German healthcare workers (66)	Higher spike-specific CD8 T-cell responses than homologous regimens	Equivalent to or better than homologous BNT schedule	Safe, with pronounced reactogenicity in ChAdOx-primed individuals	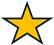
ChAdOx1 + mRNA-1273	Klemis et al., 2022 [25]	German healthcare workers (101)	Strongest T-cell response among all regimens studied	Higher antibody levels than homologous ChAdOx or BNT	Safe, but most reactogenic combination tested	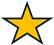
Ad26.COV2.S + mRNA-1273	Natarajan et al., 2022 [23]	US adults (25,244)	Heterologous boosting significantly improved neutralizing antibody response	78% against hospitalization vs. 67% with homologous	Safe, but higher reactogenicity than homologous boosting	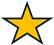
Heterologous Mix (Pfizer + Moderna, AstraZeneca + Pfizer)	Palanica et al., 2022 [28]	Canadian survey (1002)	N/A	Higher side effects reported with Moderna second dose	Safe, but concerns over long-term effects noted by participants	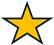
Pfizer + AstraZeneca (heterologous boost)	Awadalla et al., 2025 [41]	Saudi Arabia (484)	Higher IgG antibody levels one year post-vaccination; enhanced T-cell response (CD8+ IFN-γ production)	Superior ACE2-binding inhibition against Omicron variants; effective across all dose regimens	Safe; heterologous regimens showed longer-lasting immune durability	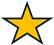

Homologous and heterologous immunization are comparable; heterologous immunization is superior. Improvement is determined based on adaptive immune response and/or clinical outcomes.

## Data Availability

No new data were created or analyzed in this study. Data sharing is not applicable to this article.
